# Ethnic differences in the association between depression and chronic pain: cross sectional results from UK Biobank

**DOI:** 10.1186/s12875-015-0343-5

**Published:** 2015-10-06

**Authors:** Barbara I. Nicholl, Daniel J. Smith, Breda Cullen, Daniel Mackay, Jonathan Evans, Jana Anderson, Donald M. Lyall, Chloe Fawns-Ritchie, Andrew M. McIntosh, Ian J. Deary, Jill P. Pell, Frances S. Mair

**Affiliations:** Institute of Health & Wellbeing, University of Glasgow, Glasgow, UK; Centre for Cognitive Ageing and Cognitive Epidemiology, Department of Psychology, University of Edinburgh, Edinburgh, UK; Division of Psychiatry, University of Edinburgh, Edinburgh, UK

**Keywords:** Chronic pain, Depression, Ethnicity, Comorbidity

## Abstract

**Background:**

Comorbid chronic pain and depression is a challenging dyad of conditions to manage in primary care and reporting has shown to vary by ethnic group. Whether the relationship between depression and chronic pain varies by ethnicity is unclear. This study aims to explore chronic pain and depression reporting across ethnic groups and examine whether this association differs, independently of potential confounding factors.

**Methods:**

Cross-sectional study of UK Biobank participants with complete data on chronic pain and probable lifetime history of depression, who reported their ethnic group as White, Asian/Asian British or Black/Black British. Chronic pain classification: present if participants had ≥ 1 site of body pain (up to seven sites or “pain all over the body” could be selected) that lasted ≥ 3 months; extent of chronic pain categories: 0, 1, 2–3, 4–7 sites or pain all over the body. Probable depression classification: an algorithm of low mood, anhedonia and help-seeking behaviour. Relationship between depression and presence/extent of chronic pain assessed using logistic/multinomial regression models (odds ratio (OR); relative risk ratio (RRR), 95 % confidence intervals), adjusted for sociodemographic, lifestyle, and morbidity factors; and a final adjustment for current depressive symptoms.

**Results:**

The number of participants eligible for inclusion was 144,139: 35,703 (94 %) White, 4539 (3 %) Asian, and 3897 (3 %) Black. Chronic pain was less (40.5 %, 45.8 %, 45.0 %, respectively) and depression more (22.1 %, 12.9 %, 13.8 %, respectively) commonly reported in White participants than Asian and Black participants. Statistically significant associations between depression and presence/extent of chronic pain persisted following adjustment for potential confounding variables; this relationship was strongest for Black participants (presence of chronic pain: OR 1.86 (1.52, 2.27); RRR 1 site 1.49 (1.16, 1.91), 2–3 sites 1.98 (1.53, 2.56), 4–7 sites 3.23 (2.09, 4.99), pain all over the body 3.31 (2.05, 5.33). When current depressive symptoms were considered these relationships were attenuated.

**Conclusions:**

Chronic pain and depression reporting varies across ethnic groups. Differences in health seeking behaviour between ethnic groups may impact on the results reported. Clinicians, particularly in primary care, need to be aware of the cultural barriers within certain ethic groups to expressing concern over mood and to consider their approach accordingly.

## Background

Chronic musculoskeletal pain and depression are common, complex disorders that are challenging to treat and manage in primary care. They often co-occur and long-standing debate has surrounded the temporal nature of the relationship between chronic pain and depression and on the causes and consequences of this relationship [1]. Factors affecting the presentation and management of symptoms are wide-ranging and will involve a combination of demographic, personal, lifestyle, environmental and social elements.

Population-based studies have shown that self-reported musculoskeletal pain is more prevalent among ethnic minority groups in the UK, particularly widespread pain at multiple sites of the body [2–4]. In the US, the prevalence of self-reported pain is higher in Hispanic and Black ethnic groups than non-Hispanic White individuals [5]; however, this difference was not apparent after adjusting for potential confounders such as sociodemographic status, comorbidity, and psychological distress. Minority ethnic groups in US pain clinic populations have also reported more severe or disabling pain than the non-Hispanic White population in the US [6, 7]. Recent studies in the US have focused on trying to better explain the racial and ethnic disparities in pain treatment and management, and suggest that access to healthcare, and patient attitudes and behaviours may play a role in explaining some of the disparities observed [8–10]. Similarly, ethnic minority groups have also been reported to be less likely to seek professional help for mood problems [11] yet the prevalence of depression is reported to be higher in these groups in the UK than in the majority White ethnic group [12, 13].

Few studies have specifically considered the comorbidity of chronic pain and depression across ethnic groups, rather ethnicity has been considered as a covariate in the relationship between pain and depression or as a predictor of pain and depression [14]. In a study of 866 participants, Miller and Cano (2009) found that African Americans were almost twice as likely to have comorbid depression and pain as Caucasian Americans, relative to those with no pain or depression [15]. However, it is unclear whether similar patterns would be observed amongst other ethnic groups and whether the relationship between depression and pain is similar in US and UK ethnic groups.

Chronic pain and depression have also been shown to be some of the most common comorbidities in individuals with other physical and mental long-term conditions [16–18]. Given the ethnic [19, 20] and sociodemographic [16] gradient evident in the pattern of long-term condition reporting it is important to study the relationship between socioeconomic status, ethnicity, pain, and depression independently of other physical long-term conditions, and furthermore understand whether a history of depression or current depressive symptoms are more important. Developing an understanding of these relationships has the potential to impact on how health professionals manage chronic pain in primary care, particularly for ethnic minority groups that may be less likely to present with their low mood problems.

The overall aim of this study is to determine: 1) the prevalence of chronic pain and depression by ethnic group; and two) whether the relationship between depression and chronic pain varies by ethnicity, using data from UK Biobank. Here we report on differences in the prevalence of chronic pain and depression, and in the association between depression and pain across ethnic groups, independently of potential confounding factors, including sociodemographic, lifestyle, and morbidity factors.

## Methods

This study utilises baseline cross-sectional data from UK Biobank (http://www.ukbiobank.ac.uk/). UK Biobank is a large cohort study of over 500,000 persons aged 40–70 years across England, Scotland, and Wales. Individuals registered with the National Health Service and living within a 25 mile radius of one of 22 UK Biobank assessment centres were invited to participate [21]. Participation involved completion of touch-screen questionnaires and nurse-led interviews to collect epidemiological data on demographic, health, environmental and lifestyle factors. Questions on mood disorder were added part-way during recruitment and the 172,745 participants who completed the detailed questions on lifetime features of mood disorders were eligible for inclusion in this study.

### Ethnic groups

Participants were asked in the touch screen questionnaire “What is your ethnic group?” Response options were: White, Mixed, Asian or Asian British, Black or Black British, Chinese, and other ethnic group. Due to insufficient numbers in the other ethnic groups, only those who considered themselves as White, Asian or Asian British (including Indian, Pakistani, Bangladeshi and “any other Asian background”), and Black or Black British (including Caribbean, African and “any other Black background”) were included in this study; these groups will be referred to in this manuscript as White, Asian, and Black ethnic groups.

### Classification of chronic pain

Within UK Biobank, participants were asked whether they had experienced pain in the past month that interfered with their usual activities, in any of the following sites: headache, facial, neck or shoulder, back, stomach or abdominal, hip, and knee; and an eighth option of “pain all over the body”. Multiple sites could be selected, unless participants reported “pain all over the body”. For all sites selected a subsequent question asked whether the pain had been present for more than three months. Chronic pain was defined as pain that had persisted for more than three months. The presence of chronic pain was classified if participants reported any site of chronic pain; those reporting no sites of chronic pain were classified as being free of chronic pain. The extent of chronic pain was calculated by summing the number of sites of chronic pain reported and the following categories were defined: free of chronic pain; chronic pain at one site; chronic pain at 2–3 sites; chronic pain at 4–7 sites; and “pain all over the body”. This classification of number of pain sites has previously been reported [22].

### Classification of depression

**Fig. 1:**
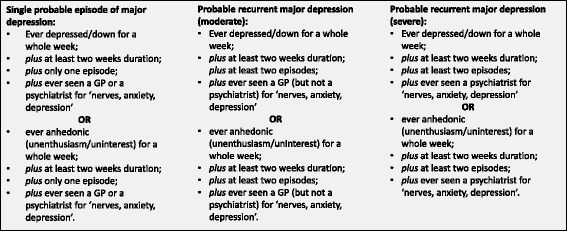
Detailed description of the classification of a probable lifetime history of depression using available data fields in UK Biobank; a positive classification for probable lifetime depression included all three depression categories: single, recurrent (moderate), and recurrent (severe)

The definition of probable lifetime depression history among UK Biobank participants has been described in detail previously [23]. Using the touch screen questionnaire, participants answered questions regarding their past history of mood disorder symptoms and related medical help-seeking, which were similar to questions within the Structured Clinical Interview for DSM-IV Axis I Disorders [24]. Probable major depressive disorder included participants that we classified as having a lifetime history of recurrent severe, recurrent moderate or single episode depression; the criteria for these definitions are outlined in Fig. 1. Current depressive symptom score was calculated from four individual questions that assessed the frequency of the following depression symptoms “over the past 2 weeks”: depressed mood, unenthusiasm/disinterest, tenseness/restlessness, and tiredness/lethargy. Questions were answered on a four point scale: not at all; several days; more than half the days; and nearly every day. Individual answers were summed to give a score of 0–12 for each participant; with higher scores reflecting greater levels of current depressive symptoms. This score was independent of the classification of lifetime depression status described above and in Fig. 1.

### Potential confounding variables

Sociodemographic, lifestyle and morbidity factors are associated with both depression and chronic pain. Alongside age and sex, Townsend Score, an area level measure of deprivation based on the participant’s postcode of residence [25], was used to describe the sociodemographic profile of the study population. Quintiles of the Townsend score were generated for the study population; quintile 1 represents the least deprived and quintile 5 the most deprived areas. Current smoking status (never, current, or former smokers) and frequency of alcohol consumption (daily/almost daily, 3–4 times per week, 1–2 times per week, 1–3 times per month, special occasions only and never) were self-reported. Body mass index (BMI) was calculated from height and weight measurements taken at the assessment centre and categorised as underweight (<18.5 kg/m^2^), normal weight (18.5–24.9 kg/m^2^), overweight (25.0–29.9 kg/m^2^), and obese (≥30.0 kg/m^2^) to describe the population but was treated as a continuous variable in the regression analysis. A count of self-reported, non-cancer long-term conditions was generated from the information provided during the nurse interview. Inclusion of conditions was based on previous literature [16], author experience (FM) and a UK Biobank prevalence of ≥0.1 %. Painful and psychiatric conditions were excluded from the count for this study, resulting in 36 conditions being included in the count.

### Ethical approval

Electronic, informed consent was given in person at the assessment centre before participation in the study. This study forms part of UK Biobank project 7155 and UK Biobank has full ethical approval from NHS National Research Ethics Service (approval letter dated 17 June 2011, Ref 11/NW/0382).

### Statistical analysis

Chronic pain, depression, and comorbid pain and depression prevalence are reported for each ethnic group, along with sociodemographic, lifestyle and morbidity variables. Chi squared tests for categorical variables and Kruskal Wallis tests for continuous variables are used to assess differences between ethnic groups for each of the pain, depression and potential confounding variables. To quantify the association between depression and the presence of chronic pain, logistic regression models were used; results are presented as odds ratios (OR) and 95 % confidence intervals. To quantify the association between depression and the extent of chronic pain reported, multinomial logistic regression models were used; results are presented as relative risk ratios (RRR) and 95 % confidence intervals. The association between depression (independent variable) and chronic pain (dependent variable) was quantified using a separate regression model for each ethnic group as we were specifically interested in the relationship between depression and chronic pain within ethnic groups. Interaction between depression and ethnicity with both chronic pain outcomes was assessed and was indicated for some sub-groups at approximately *p* = 0.10. For both chronic pain outcomes (presence and extent of chronic pain), levels of adjustment for the regression models were as follows: 1) univariate analysis (no adjustment); 2) adjusted for sociodemographic variables – age, sex and quintiles of Townsend score; 3) adjusted for sociodemographic and lifestyle variables – as in 2) plus smoking status, frequency of alcohol consumption and BMI; 4) adjusted for sociodemographic, lifestyle and morbidity count – as in 3) – plus number of long-term conditions reported. In order to investigate the impact of current depressive symptom score as this may impact on an individual’s current pain reports (and vice versa), a final adjustment for current depressive symptom score is described in the text. All analysis was conducted using Stata V13.0 [26]. The number of participants included in each model varied according to the proportion of missing data for each of the potential confounding variables; however, the completion rate of confounding variables included in the regression models was at least 99.3 %, with the exception of current depressive symptom score, which was completed by 92.0 %.

## Results

Of the 172,743 UK Biobank participants who were asked questions on lifetime history of depression and manic symptoms, 28,604 (16.6 %) were excluded: 22,901 (13.3 %) provided incomplete data precluding classification of their lifetime history of mood disorder features; 1615 (0.9 %) had probable bipolar disorder; 3876 (2.2 %) were not in the three main ethnic groups (or did not provide data on their ethnicity); and 212 (0.1 %) did not provide complete data on pain. Thus, 144,139 participants were included in the study: 135,703 (94.1 %) were White, 4539 (3.1 %) Asian, and 3897 (2.7 %) Black. Of the 144,139 participants, 40.8 % reported chronic pain in one or more body sites and 21.6 % were classified as having probable lifetime history of depression; comorbid chronic pain and depression was present in 10.9 % of included participants (Table 1).

**Table 1:** Prevalence of chronic pain and depression overall and by ethnic group

		Ethnic group^a^		
	Overall *N* = 144,139	White *N* = 135,703	Asian *N* = 4539	Black *N* = 3897
Presence of chronic pain^b^				
Yes *N (%)*	58,785 (40.8)	54,949 (40.5)	2081 (45.8)	1755 (45.0)
Extent of chronic pain^b^				
0 *N (%)*	85,354 (59.2)	80,754 (59.5)	2458 (54.2)	2142 (55.0)
1	32,948 (22.9)	31,040 (22.9)	1023 (22.5)	885 (22.7)
2–3	20,975 (14.6)	19,554 (14.4)	791 (17.4)	630 (16.2)
4–7	3371 (2.3)	3096 (2.3)	145 (3.2)	130 (3.3)
Pain all over the body	1491 (1.0)	1259 (0.9)	122 (2.7)	110 (2.8)
Depression (lifetime history)^b^				
Yes *N (%)*	31,080 (21.6)	29,957 (22.1)	584 (12.9)	539 (13.8)
Depression and chronic pain status^b^				
Free of depression and chronic pain *N (%)*	69,942 (48.5)	65,795 (48.5)	2214 (48.8)	1933 (49.6)
Depression only	15,412 (10.7)	14,959 (11.0)	244 (5.4)	209 (5.4)
Chronic pain only	43,117 (29.9)	39,951 (29.4)	1741 (38.4)	1425 (36.6)
Chronic pain and depression	15,668 (10.9)	14,998 (11.1)	340 (7.5)	330 (8.5)
Sex^b^				
Female *N (%)*	79,596 (53.1)	72,166 (53.2)	2122 (46.8)	2308 (59.2)
Age (years)^b^				
*Mean (SD)*	56.8 (8.1)	57.1 (8.1)	53.6 (8.6)	52.3 (8.0)
Townsend Index quintiles				
Missing data *N*	230	210	10	10
1 (least deprived) *N (%)*	24,482 (17.0)	24,091 (17.8)	304 (6.7)	87 (2.24)
2	29,077 (20.2)	28,557 (21.1)	351 (7.8)	169 (4.4)
3	29,978 (20.8)	28,959 (21.4)	674 (14.9)	345 (8.9)
4	32,347 (22.5)	29,760 (22.0)	1575 (34.8)	1012 (26.0)
5 (most deprived)	28,025 (19.5)	24,126 (17.8)	1625 (35.9)	2274 (58.5)
Smoking status				
Missing data *N*	445	389	35	21
Never *N (%)*	80,000 (55.7)	73,679 (54.5)	3544 (78.7)	2777 (71.7)
Former	53,717 (37.4)	52,184 (38.6)	718 (15.9)	815 (21.0)
Current	9977 (6.9)	9451 (7.0)	242 (5.4)	284 (7.3)
Frequency of alcohol intake				
Missing data *N*	66	44	15	7
Daily/almost daily *N (%)*	30,118 (20.9)	29,530 (21.8)	335 (7.4)	253 (6.5)
3–4 times/week	33,362 (23.2)	32,552 (24.0)	412 (9.1)	397 (10.2)
1–2 times/week	36,663 (25.5)	35,204 (30.0)	664 (14.7)	795 (20.4)
1–3 times/month	16,190 (11.2)	15,239 (11.2)	378 (8.4)	573 (14.7)
Special occasions only	16,376 (11.4)	14,388 (10.6)	876 (19.4)	1112 (28.6)
Never	11,364 (7.9)	8745 (6.5)	1859 (41.1)	760 (19.5)
BMI				
Missing data *N*	980	765	126	89
* Mean (SD)*	27.5 (1.8)	27.4 (4.7)	27.0 (4.4)	29.6 (5.4)
Morbidity count^b^				
* Mean (SD)*	0.9 (1.0)	0.8 (1.0)	0.9 (1.1)	0.9 (1.0)
Current depressive symptom score				
Missing data *N*	11,511	10,018	833	660
* Mean (SD)*	1.5 (2.0)	1.4 (1.9)	2.5 (2.8)	2.2 (2.7)

*SD* Standard deviation; *BMI* Body mass index

^a^Differences between groups were statistically significant at *p* < 0.001 for all variables (tested by Chi^2^ test for categorical variables and Kruskal Wallis test for continuous variables)

^b^No missing data

### Description of ethnic groups

Over half the participants in all three ethnic groups considered in this study reported no chronic pain (54–60 %) and approximately 23 % in all groups reported only one site of chronic pain. A greater proportion of Asian and Black participants reported multisite pain (2–3 and 4–7 sites) and pain all over the body compared to White participants (Table 1). A more apparent difference in prevalence of lifetime history of depression was observed across the three ethnic groups. A significantly greater proportion of the White ethnic group were classified as having a lifetime history of probable depression compared to the Asian and Black ethnic groups (22.1 %, 12.9 % and 13.8 %, respectively). Eleven percent of White participants reported depression without pain compared to only 5 % of the Asian and Black groups. In contrast, 38.4 % and 36.6 % of Asian and Black participants respectively reported chronic pain without depression, compared with 29.4 % of White participants. Although comorbid chronic pain and depression was more common in the White ethnic group (11.1 %) it was the same proportion as that reporting depression only in this group. Whereas in the Asian and Black groups, a greater proportion reported comorbid chronic pain and depression than depression alone.

Table 1 also summarises ethnic groups by demographic, lifestyle and morbidity factors. The Asian and White groups included a lower proportion of females than the Black ethnic group. A greater proportion of both the White and Black ethnic groups reported being former or current smokers and drinking alcohol than the Asian ethnic group. A greater proportion of the Asian and Black groups were classified in the most deprived quintile compared to the White group; these groups were also younger than the White group. BMI was noticeably higher in the Black ethnic group compared to Asian and White groups and current depressive symptom score was noticeably higher in the Asian and Black groups than the White group.

### Association between depression and the presence of chronic pain

In the univariate analysis for all three ethnic groups, having depression was associated with at least a 65 % increased odds of reporting chronic pain; although this relationship was greater in Asian and Black ethnic groups. Adjusting for demographic factors in model two had little effect on these relationships (see Table 2). Further adjustment to include lifestyle factors did decrease the strength of the relationship between depression and chronic pain reporting, however, the relationships remained significant. Fully adjusting the models for demographic and lifestyle factors, and number of morbidities, attenuated the relationships further but a significant association between depression and the presence of chronic pain remained for all three ethnic groups, and was strongest in the Asian and Black ethnic groups: White (OR = 1.46, 95 % CI 1.43, 1.51), Asian (OR = 1.53, 95 % CI 1.27, 1.85), and Black ethnic group (OR = 1.86, 95 % CI 1.52, 2.27).

**Table 2:** Logistic regression analysis, stratified by ethnic group, of the relationship between depression and the presence of chronic pain

	Ethnic group		
Level of adjustment^a^	White OR (95 % CI)*	Asian OR (95 % CI)*	Black OR (95 % CI)*
Model 1 - no adjustment	1.65 (1.61, 1.69)	1.77 (1.48, 2.11)	2.14 (1.78, 2.58)
Model 2 - adjusted for demographic factors	1.61 (1.57, 1.66)	1.71 (1.43, 2.05)	2.12 (1.75, 2.56)
Model 3 - adjusted for demographic and lifestyle factors	1.53 (1.45, 1.57)	1.62 (1.35, 1.95)	1.99 (1.63, 2.42)
Model 4 - adjusted for demographic, lifestyle and morbidity count	1.46 (1.43, 1.51)	1.53 (1.27, 1.85)	1.86 (1.52, 2.27)

*All individual associations and the overall models were significant at *p* < 0.001

^a^Referent group for all models is the free of chronic pain group

### Association between depression and the extent of chronic pain

For all three ethnic groups, the RRR between depression and chronic pain in model one (unadjusted) increased as the number of pain sites reported increased. The strength of the relationship between depression and chronic pain reporting was more apparent in both Asian and Black ethnic groups than in the White ethnic group, when 2–3 pain sites, 4–7 pain sites, or pain all over the body was reported. This pattern of association remained through all levels of adjustment, although the RRRs were attenuated (Table 3). In the fully adjusted model (model 4) a dose response relationship between depression and extent of pain reported was evident for Asian and Black ethnic groups. In the White ethnic group this dose response was evident up to 4–7 sites of pain and then leveled off for “pain all over the body”. The RRR of reporting 4–7 sites of pain was 2.35 (95 % CI 2.17, 2.54) in the White ethnic group, 2.63 (95 % CI 1.67, 4.13) in the Asian ethnic group, and 3.31 (95 % CI 2.05, 5.33) in the Black ethnic group.

**Table 3:** Multinomial logistic regression analysis, stratified by ethnic group, of the relationship between depression and the extent of chronic pain

		Ethnic group		
Level of adjustment^	Extent of chronic pain	White RRR (95 % CI)	Asian RRR (95 % CI)	Black RRR (95 % CI)
Model 1 – no adjustment	1 pain site	1.36 (1.32, 1.40)	1.25 (0.99, 1.57)**	1.58 (1.25, 2.00)
	2–3 pain sites	1.89 (1.82, 1.95)	1.97 (1.57, 2.47)	2.29 (1.80, 2.92)
	4–7 pain sites	3.16 (2.94, 3.40)	3.46 (2.35, 5.09)	4.26 (2.87, 6.33)
	Pain all over the body	2.96 (2.64, 3.31)	3.65 (2.41, 5.52)	4.32 (2.82, 6.61)
Model 2 - adjusted for demographic factors	1 pain site	1.35 (1.31, 1.39)	1.22 (0.97, 1.54)**	1.61 (1.27, 2.05)
	2–3 pain sites	1.82 (1.76, 1.89)	1.92 (1.53, 2.41)	2.23 (1.74, 2.86)
	4–7 pain sites	2.94 (2.73, 3.17)	3.30 (2.22, 4.90)	4.18 (2.78, 6.29)
	Pain all over the body	2.80 (2.49, 3.14)	3.60 (2.37, 5.48)	4.05 (2.61, 6.29)
Model 3 - adjusted for demographic and lifestyle factors	1 pain site	1.31 (1.27, 1.36)	1.16 (0.92, 1.47)**	1.52 (1.19, 1.95)*
	2–3 pain sites	1.72 (1.66, 1.78)	1.82 (1.47, 2.34)	2.11 (1.64, 2.73)
	4–7 pain sites	2.66 (2.47, 2.87)	3.02 (2.00, 4.58)	3.63 (2.36, 5.58)
	Pain all over the body	2.48 (2.20, 2.79)	3.23 (2.08, 5.00)	4.10 (2.59, 6.50)
Model 4 – adjusted for demographic, lifestyle and morbidity count	1 pain site	1.29 (1.24, 1.33)	1.14 (0.90, 1.45)**	1.49 (1.16, 1.91)*
	2–3 pain sites	1.63 (1.57, 1.69)	1.77 (1.40, 2.24)	1.98 (1.53, 2.56)
	4–7 pain sites	2.35 (2.17, 2.54)	2.52 (1.65, 3.87)	3.23 (2.09, 4.99)
	Pain all over the body	2.19 (1.94, 2.47)	2.63 (1.67, 4.13)	3.31 (2.05, 5.33)

All individual associations were significant at *p* < 0.001 except for *p ≤ 0.002 and **p > 0.05

Overall models were significant at *p* < 0.001

^ Referent group for all models is the free of chronic pain group

### Impact of current depressive symptoms

Including current depressive symptom score in a final model greatly attenuated the relationship between probable depression and both the presence and extent of chronic pain for all three ethnic groups. There was no longer a statistically significant relationship between probable depression and presence/extent of chronic pain in Asian participants. However, a statistically significant relationship remained between depression and presence of chronic pain for both the White (OR = 1.22, 95 % CI 1.19, 1.27) and Black (OR = 1.41, 95 % CI 1.13, 1.78) ethnic groups, and a similar pattern to model 4 (Table 3) was observed with the extent of chronic pain for these groups.

## Discussion

Our study has demonstrated that chronic pain in at least one body site is very common, affecting more than 40 % of our UK Biobank study population. Furthermore over one fifth of the population were classified as having a probable lifetime history of depression, and comorbid chronic pain and depression was present in 11 % of included participants. Chronic multisite pain was reported more commonly and lifetime history of probable depression less commonly in Black and Asian ethnic groups in UK Biobank compared to the majority White study population. Quantification of the association between depression and chronic pain suggests that the relationship between depression and both the presence and extent of chronic pain is stronger in the minority ethnic groups, particularly the group comprising those of Black ethnicity. These findings suggest that although depression is less common in minority ethnic groups it is more strongly associated with chronic pain, particularly in Black ethnicities. However, on a formal test of the coefficients in model 4 (*suest* command in Stata), the difference between ethnic groups was not statistically significant for the presence or extent of chronic pain, except for the difference between the presence of chronic pain in Black compared to White ethnic groups.

This study resonates with previous reports from much smaller study populations of an increased prevalence of chronic pain, including multiple sites of joint pain and widespread pain, in UK minority ethnic groups [2–4]. We have also shown that there is a dose-response relationship in the association between depression and the number of sites of pain reported across all ethnic groups, although this pattern appears to be more marked for the minority ethnic groups studied. To our knowledge this is the first study to have reported this relationship. The pattern of the association between depression and “pain all over the body” was not consistent across ethnic groups. Participants in Asian and Black ethnic groups were three times more likely to report “pain all over the body” than White participants. Replication in a larger sample of participants from minority ethnic groups with “pain all over the body” would be valuable. Miller and Cano reported (based on 80 participants) that African Americans in the United States were more likely to report comorbid depression and chronic pain than a non-Hispanic White group [15]. However, in the current study, comorbid chronic pain and depression reporting was more common in the White population, which was a similar proportion to that which reported depression only in this group (11 %). In contrast, in the Asian and Black groups, a greater proportion reported comorbid chronic pain and depression (8 and 9 %, respectively) than depression alone (5 % for both groups), suggesting that ethnic minority groups in the UK are more likely to report depression alongside their pain rather than depression alone, showing some similarity with the American study.

We have also shown that although probable lifetime depression was reported less commonly in minority ethnic groups in this study; these groups did report a higher mean current depressive symptom score compared to the White ethnic group. Including current depressive symptom score in the fully adjusted regression models attenuated the relationship between probable depression and presence/extent of chronic pain in White and Black ethnic groups, and completely diminished the relationship in the Asian ethnic group. Our definition of probable lifetime depression was based on a relatively strict definition which included help-seeking behaviour as a key criterion (Fig.1) [23]. It is therefore quite possible that individuals from minority ethnic groups in the UK are aware of symptoms of low mood but may not have sought medical help for these and may be more likely to seek help for physical symptoms. Furthermore there is evidence to suggest that mental health problems in Black and minority ethnic groups are less likely to be detected by a GP [27, 28]. It could be hypothesised that somatic symptoms included in the current depressive score (tenseness/restlessness and tiredness/lethargy) may be more highly endorsed than the psychological symptoms of low mood and anhedonia by individuals in the minority ethnic groups, yet closer inspection revealed that all items are endorsed as experienced on “more than half the days” or “nearly every day” over the past two weeks more frequently by participants from the Asian and Black ethnic groups than the White ethnic group. However, this tool to assess current depressive symptoms has not been validated and the items could potentially function differently across the three ethnic groups in this study.

### Strengths and Limitations

Major strengths of this study include the large scale of the UK Biobank dataset of middle-aged to older adults in England, Scotland, and Wales. We conducted this analysis on a large subset of the UK Biobank population, which included over 3000 participants from both Asian and Black ethnic groups. Furthermore, participation in UK Biobank involved detailed assessment of a large number of demographic, lifestyle and morbidity factors, which allowed the association between depression and chronic pain (presence and extent) to be considered independently of potential confounders, including number of comorbid physical long-term conditions. Multimorbidity has been shown to be more common in minority ethnic groups [20]; our results suggest that the relationship between depression and pain is independent of other physical long-term conditions across all ethnic groups although we were unable to consider the functional or physical limitations caused by such conditions, which has been shown to be important [13]. There are further limitations to this study. The information included in this study (except BMI) was self-reported. This has potential implications for the reliability of the data; however, the large sample size suggests it is unlikely that over or under reporting of chronic pain or depressive symptoms would impact on the results observed. The validity of our classification of a lifetime history of probable depression has previously been considered in detail [23] and shown to be distributed as expected by socioeconomic status, gender, self-reported health, and smoking. Our study was cross-sectional in nature and we are therefore unable to determine the temporal relationship between pain and depression reporting in this group. Due to the smaller number of participants from “mixed” (*N* = 1062), Chinese (*N* = 534) and “other ethnic group” (*N* = 1719), participants from these groups were excluded from the current study as no meaningful conclusions could have been drawn. It is known that individuals from minority ethnic groups are less likely to take part in medical research [29, 30]; however, it is unclear from UK Biobank how many individuals from minority ethnic groups were invited to take part and did not respond. The 2011 England and Wales census reported the population to be 86 % White; 7.5 % Asian or Asian British and 3.3 % Black or Black British [31]. Our study population comprised 94 % White, 3 % Asian or Asian British, and 3 % Black or Black British. We have applied exclusion criteria based on ethnicity to our study population and therefore cannot make a direct comparison between our study population and the UK population.

Our study is lacking information on the severity of the pain reported by participants. Increasing pain severity has been shown to correlate with an increased number of sites of chronic pain [32] and in our study we have shown that participants of Asian or Black ethnicity are more likely to report multisite chronic pain, suggesting that they may be experiencing more severe chronic pain. Another limitation is our lack of information on; acculturation, the extent to which ethnic groups and individuals change their culture as a result of migration [33], which may affect beliefs and actions and influence reporting of health issues. This has been demonstrated previously by Choudhury et al [4] who reported that in a study of 1223 individuals (50 % White) chronic pain and chronic widespread pain were more prevalent in the group who identified as being Bangladeshi (72 and 16 %, respectively) compared to both White (56 and 10 %, respectively) and British Bangladeshi (54 and 9 %, respectively), living in a borough of London, England. Without information on acculturation it may be that our estimates of chronic pain prevalence are an under-estimation of that in ethnic groups who do not identify as being British.

### Clinical implications

In the present study we were interested, not only in differences in the prevalence and type of pain reporting across ethnic groups, but also in the relationship between depression and chronic pain reporting; and although we found only limited statistical differences between ethnic groups for the relationship between depression and chronic pain, differences in pain and depression reporting across the groups were observed. Our findings have implications for patient management, especially in primary care. Differences in coping strategies, language barriers and the cultural interpretation of pain and low mood may influence a person’s likelihood to self-report symptoms or seek medical help, and may affect how they present in the clinical encounter, making it more difficult for health professionals to recognise these problems.

Ethnicity adds a further complexity to the already clinically complex dyad of pain and depression. It is clear, however, that early professional medical advice is helpful and further consideration of a package of treatment for the comorbid conditions is required [1] but our findings suggest that attention should be paid to whether different approaches may be more suitable for certain ethnic groups. Ethnic disparities in the management of chronic pain have received much attention in the US and studies suggest that better strategies need to be implemented to reduce these inequalities [8–10].

## Conclusions

 Cultural differences and language deficits can be barriers to good care provision for minority ethnic groups but so too can health professionals’ poor understanding of these barriers and of potential ethnic differences in modes of presentation. A key question for clinicians is how to best respond to these issues? An increased awareness of cultural differences and their potential implications for management is essential. Bhui and Bhugra (2004) suggest culturally grounded explanations of mental illness are needed [34]. This is especially important since healthcare providers are increasingly dealing with ever more ethnically diverse populations and this is likely to increase as time goes on as there are approximately 214 million migrants worldwide [35]; with 73 million currently residing in the WHO European region [36]. However, further research is needed to examine how these ethnic differences in chronic pain and depression reporting affect long term health outcomes and to explore whether there are differences in health care utilisation and the type of care accessed.

## Abbreviations

BMI: Body mass index
CI: Confidence interval
OR: Odds ratio
RRR: Relative risk ratio
SD: Standard deviation
